# Correlates of social participation of children with special healthcare needs: baseline of the PART-CHILD study

**DOI:** 10.1186/s12887-026-07233-3

**Published:** 2026-06-29

**Authors:** Carolina Fioroni Ribeiro da Silva, Angélique Herrler, Marielle Wirth, Simone Weyers, Freia De Bock

**Affiliations:** 1 Institute of Medical Sociology, Centre for Health and Society, Medical Faculty and University Hospital Düsseldorf, Heinrich Heine University, Düsseldorf, Germany; 2 Department of General Pediatrics, Neonatology and Pediatric Cardiology, Medical Faculty and University Hospital Düsseldorf, Heinrich Heine University, Düsseldorf, Germany; 3 Institute of Medical Sociology and Rehabilitation Science, Charité – Universitätsmedizin Berlin, Corporate Member of Freie Universität Berlin and Humboldt-Universität zu Berlin, Berlin, Germany; 4 Institute for Biometrics and Epidemiology, German Diabetes Center (DDZ), Leibniz Center for Diabetes Research at Heinrich Heine University Düsseldorf, Düsseldorf, Germany

**Keywords:** Health-related quality of life, Impairment, Physical, Cognitive, School, Community, Home, Child and Adolescent Scale of Participation (CASP), Social participation

## Abstract

**Background:**

Children with special healthcare needs (CSHCN) live with long-term physical, developmental, behavioural, or mental conditions that affect everyday functioning and require specialized care. According to the International Classification of Functioning, Disability and Health (ICF), such conditions may cause impairments in body functions and structures, limitations in activities, and restrictions in participation, shaped by environmental factors. Evidence on social participation and its correlates in CSHCN remains limited. Therefore, we aimed to identify which correlates are associated with social participation across different conditions and indications in a multi-site sample of CSHCN in Social Paediatric Centres (SPCs) in Germany.

**Methods:**

This study analysed parent-reported data of CSHCN (3–18 years) from the baseline of the PART-CHILD cohort collected in routine care in 15 SPCs in Germany (2019–2020). Social participation (outcome) in home, community, school, and living activity domains was assessed using the German Child and Adolescent Scale of Participation (CASP). In the regression analysis, independent variables included environmental factors (parental educational attainment, German native language) and health-related factors (impairment type and complexity reported as reason for referral to the SPCs, health-related quality of life (HRQoL)), as well as age (operationalised in groups [3–6; 7–10; 11–18 years]) and sex as covariates. Linear complete case regression models were performed, and β-coefficients and 95% confidence intervals were reported.

**Results:**

Five hundred fifty-one families of CSHCN (68.24% female; 44.46% aged three to six and 34.66% from seven to ten years old) were available for analysis (10.99% missing outcome). CASP total mean score was 75.99 (± 20.03; range: 25–100), the lowest in living activities subscale (68.07 ± 24.81), including household activities, shopping, transportation. In the complete case regression analysis (*n* = 350), older child age and higher HRQoL were associated with higher social participation score across all domains. Children with both physical and cognitive impairments had lower CASP scores in the total, home, and school models, with a trend to association in the community model. Lower parental educational attainment was significantly associated with reduced social participation in the living activities subscale.

**Conclusions:**

In this study, we confirmed previous findings on factors determining social participation in CSHCN, such as age and higher HRQoL. Impairment complexity (combined physical and cognitive impairments) decreased participation in home and school. The lower participation in daily living activities for lower educated parents confirms, that social environment significantly influences social participation. This highlights that to improve social participation, strategies are needed that address family-level *and* broader environmental barriers to lessen social and functional disadvantages in particularly vulnerable subgroups.

**Trial registration:**

PART-CHILD cohort, German Clinical Trials Register - Deutsches Register Klinischer Studien (DRKS), DRKS00015054, registered on 16 November 2018.

**Supplementary Information:**

The online version contains supplementary material available at 10.1186/s12887-026-07233-3.

## Background

Children with special healthcare needs (CSHCN) are defined as those who have physical, developmental, behavioural, or emotional conditions, such as asthma, cerebral palsy, attention-deficit and hyperactivity disorder (ADHD), or genetic disorders [1]. These conditions directly impact CSHCN’s everyday life functioning, at the same time meaning that they require more extensive and complex health services to attend their needs and support their development [1, 2].

The prevalence of CSHCN and adolescents in the Western world reported in the literature varies widely in the last years (2011 to 2024), ranging from 15.7% (Spain) [3] to 43.0% (the United States) [4–6]. These numbers may vary depending on the method used to identify special healthcare needs in each country and also to define them [7]. In Germany, data from the German Health Interview and Examination Survey for Children and Adolescents (KiGGS) showed a weighted prevalence of CSHCN of 16.0% among boys and 11.4% among girls in the period from 2003 to 2006 [8]. Because of the high prevalence and extensive support needs, CSHCN represent an important public health priority in Western world [4–6, 9]. It is a challenge to provide suitable healthcare for all CSHCN [10, 11], but particularly for school-aged children (6–11 years), who have been shown to require more continuous treatment and assistance [6] than CSHCN in other age groups. With the transition into school as a new life phase, CSHCN gain opportunities to enhance their social participation. However, the early years of schooling are also marked by a range of challenges: increasing academic and social demands, clearer recognition of developmental and learning difficulties, and rapid physical growth, necessitating ongoing therapeutic adjustments, assistive device modifications, and sustained coordination between healthcare providers, families, and educational systems, for instance, as observed in children with ADHD [12]. When CSHCN are supported by a system that addresses their social, health, and emotional needs as well as appropriate child development, they can live fulfilling lives from early childhood into adulthood [2]. However, such a system should promote their dignity, autonomy, independence and support social participation [2].

Social participation in paediatrics means that, regardless of disabilities, children are actively involved in meaningful ways in everyday social situations, such as playing games, eating at the table with their relatives, making friends, and consequently developing cognitive and motor skills [13]. It plays an essential role in the rehabilitation and neuroplasticity processes to drive experience-dependent plasticity through repeated, task-specific, and socially meaningful practice that strengthens synapses, reorganizes functional networks, and supports myelination [14], fundamental elements to the effectiveness and success of treatment [15, 16]. Social participation in addition aligns with the principles of inclusion and non-discrimination established by the UN Convention on the Rights of Persons with Disabilities [17], which also applies to CSHCN.

Evidence shows that higher participation in community, school, or sports activities is associated with better health-related quality of life (HRQoL) [18]. On the other hand, lower participation is associated with poorer outcomes, especially in those with disabilities, such as cerebral palsy [19]. For example, participation mediates the effect of functional limitations on quality of life, suggesting that interventions targeting increased engagement in social or community activities may improve other outcomes, for instance, functionality, activities and HRQoL, even when impairments persist [20, 21].

To assess social participation comprehensively, as specified in the International Classification of Functioning, Disability and Health (ICF) [22], different instruments exist [23], for instance, the Child and Adolescent Scale of Participation (CASP) [24–26] and the Participation and Environment Measure for Children and Youth (PEM-CY) [27]. Recent studies on social participation of CSHCN demonstrated by CASP and PEM-CY instruments that the type and severity of the child’s condition are associated with their social participation. For example, children with mild chronic health conditions had significantly higher levels of social participation compared to those with severe conditions (Germany) [25] and among children with neuromuscular diseases, motor impairments were moderately associated with social participation (France) [28]. Other studies using PEM-CY and CASP conducted with children with acquired brain injury (Netherlands) [29] and children with underlying health conditions and functional limitations (Canada) [30] showed that disadvantaged family circumstances, such as ethnic minority status, limited material and social resources, lower parental educational level, poor parental mental health [31], and fatigue [29] were related to restrictions in social participation. A systematic review further highlighted age, health status, and environmental factors as common barriers to social participation [31]. So far, some studies showed that CSHCN who are exposed to disadvantageous social circumstances and have more severe conditions may exhibit the lowest levels of social participation [28–31]. However, the existing evidence is heterogeneous in terms of measurement instruments, domains (Home, Community, School or preschool, and Daily living activities), samples, diagnoses and quality [31]. The literature on participation is characterized by concept and measurement variation. A systematic review identified 118 different participation measures used across studies of children with disabilities, reflecting the diversity of participation constructs and outcomes assessed [32]. In addition, there are no studies reporting social participation in German CSHCN treated in Social Paediatric Centres (SPCs) so far.

SPCs are interdisciplinary, physician-led outpatient paediatric care centres in Germany that provide routine diagnosis and treatment for CSHCN [33], and they represent an important area for clinical practice and routine care in Germany [33], caring for 466.00 CSHCN per year, i.e. approximately 3.5% of all children in Germany [34]. Routine care in SPCs refers to the usual interdisciplinary outpatient assessment, counselling, therapy planning, and follow-up for children and adolescents with chronic conditions and developmental disorders, delivered by a multidisciplinary team and embedded in regular clinical practice, including coordination with family, school/kindergarten, and other services [33].

Despite growing awareness of the importance of social participation for CSHCN, social participation in paediatric field is a relatively new concept that has only gained prominence in the past one to two decades [35] and studies which investigate social participation in CSHCN and its correlates are scarce [36]. Investigating social participation in everyday life domains, for instance, at school, home and community, and understanding the correlates to social participation might allow developing interventions focused on participation, which may be more relevant for CSHCN than interventions focused solely on impairment or body functions [37]. Also, barriers to participation could be tackled more intensely in rehabilitation, e.g., based on the biopsychosocial model, as it enables clinicians to address not only impairments but also environmental and social factors that restrict participation.

In correspondence to the biopsychosocial model, the ICF conceptualises participation as the result of dynamic and multidirectional interactions between health conditions, body functions and structures, activities, environmental factors, and personal factors [22]. Participation encompasses attendance and involvement. Attendance refers to the person’s presence (‘being there’) and can be seen as the objective component (‘formal integration into social groups’), while involvement is the subjective component (‘feeling of being included’) [38]. Within the biopsychosocial model, social participation is not understood as a consequence of diagnosis alone, but as the result of dynamic and multidirectional interactions between health conditions, body functions and structures, activities, environmental factors, and personal factors [22]. Therefore, social participation is not determined by a diagnosis alone. Rather, it emerges from the interplay of biological, psychological, and social influences, including functional abilities, environmental opportunities, barriers, and personal characteristics such as temperament and personality.

Building upon the ICF, the present study aims to identify which correlates are associated with social participation across different conditions and indications in a multi-site routine care sample of CSHCN in SPCs in Germany. Accordingly, to the biopsychosocial model and the ICF, in this study, age, HRQoL, and impairment type and complexity were considered indicators related to functioning and health status, whereas parental educational attainment and native language were considered environmental factors that may influence participation across domains.

## Methods

### Ethics approval and registration

The study was approved by the Ethics Committee of the Medical Faculty Mannheim, Heidelberg University, Germany (Ref. 2018–529 N-MA) [33]. All procedures complied with the Declaration of Helsinki, national guidelines of the German National Health Council, and the EU General Data Protection Regulation. The study was also registered in the German Clinical Trials Register (DRKS00015054). All parents, patients and staff members of SPCs signed the written informed consent as participants.

### Study design and data source

This study is based upon baseline data of the PART-CHILD cohort [33], which longitudinally measures the social participation of CSHCN in SPCs in Germany, as the first of its kind cross-indication, multi-site sample in Germany. In this study, we analysed only the data from the first wave (baseline), collected from February 4, 2019, to February 27, 2020, and performed a cross-sectional analysis, while variable selection and use was guided by the ICF framework. Further details are available in the published study protocol [33]. We reported the study following the STROBE guideline [39].

All parents of children aged 3 to 16 years having appointments in one of the 15 randomly selected German SPCs participating in the PART-CHILD Study [33] were planned to be invited to participate (between November 2018 and January 2020) by routine professional staff of the local SPC. Yet, SPC are caring for children until the age of 18 years according to health insurance contracts. Forty-one of 156 SPCs (26%) had submitted an application to participate in the study; 27/41 (66%) met all inclusion criteria and no exclusion criteria. One eligible SPCs was excluded due to a staff size of 190 and a very high patient volume. Next, the trial statistician randomly selected 15 SPCs from the eligible sites, stratified by patient volume (small: 1700–2600 referrals/3 months; medium: 3870–4934; large: 5000–12,027) and scope of services (diagnostic vs. diagnostic and treatment). The final sample comprised 3 diagnostic SPCs (one small, one medium, one large) and 12 diagnostic and treatment SPCs (four small, four medium, four large) [33, 40]. The participating SPCs were anonymised, thus it is not possible to inform the exact location of them.

The families were eligible for participation if (1) they could answer the questionnaire in German and (2) if they were expected to have at least four more appointments planned at the SPCs in the next two years [33].

Data were collected on one randomly selected day per week during the entire study period. To minimize the risk of pre-selection, study sites were notified no more than eight days in advance of each data collection day. Selection of data collection days followed a block randomization scheme conducted by the study statistician (three Mondays to Wednesdays and two Thursdays and Fridays per quarter). On data collection days, reception staff invited all eligible families to participate. After the clinical appointment, they were reminded to complete the questionnaires in the reception area. Completed questionnaires were deposited anonymously in a locked box at each study site. Given that data were collected on one random day per week and appointments occur every few weeks, repeat participation was considered unlikely. Due to logistical constraints at some sites (including lack of internet access), questionnaires were administered exclusively in paper format; an online alternative was not feasible.

### Variables

Social participation of children (dependent variable) was defined according to the ICF as the meaningful interaction between the child and their environment, influenced by individual aspects and contextual requirements [41–43], resulting in the feeling and experience of social involvedness in everyday life activities. In order to measure social participation, we used the German version of CASP [24–26]. The CASP is a freely available questionnaire that encompasses 20 items, divided in 4 scales which cover *attendance* and *involvement*, focusing more on attendance than on involvement into social circumstances of everyday life: : “home”, “community”, “school”, and “living activities subscale” (Table 1).The instrument measures the extent to which children of 3 to 18 years of age participate in activities in comparison to healthy children of the same age [25].

**Table 1:** German Child and Adolescent Scale of Participation (CASP): Subscales and Summarized Items Content

Subscale	Summary description	Summarized items
Home	Participation in family life, play, daily routines, and communication at home	(1) Social and play activities with family at home (board games, pretend play, hobbies); (2) Social and play activities with friends at home (including by phone or video); (3) Participation in family tasks and decisions (fetching items, small chores, joining family activity planning); (4) Self-care activities (eating, drinking, dressing, bathing, grooming, toileting); (5) Moving around inside and around the home (house, garden, yard); (6) Communicating with others at home (verbal, non-verbal, or using assistive communication).
Community	Participation in play, organized activities, mobility, and interactions in the neighbourhood and community	7) Social and play activities with friends in the neighbourhood (playground, park, fairs); 8) Organized community activities and events (sports clubs, youth groups, music school, seasonal festivals, theatre, cinema, restaurants); 9) Independent mobility in the neighbourhood and public places (parks, public buildings, restaurants, cinema); 10) Communicating with others in the neighbourhood (peers and adults).
School or preschool	Participation in school routines, learning activities, mobility, and communication	11) Classroom participation (raising hand, waiting turn, following rules); 12) Social and play activities at school (recess, sports, hobbies, arts and crafts, theatre, clubs); 13) Mobility across school areas (classroom, gym, playground, cafeteria, library, toilet); 14) Use of learning materials available to peers (books, computers, desks, adapted equipment if needed); 15) Communicating with peers and adults at school
Daily living activities	Independence in daily responsibilities, planning, transportation, and task completion	16) Performing household tasks (cleaning, table setting, meal preparation, laundry, dishwashing); 17) Shopping and using money; 18) Managing daily schedule independently (completing tasks, organizing and adapting routine);19) Using transportation for daily activities (to and from school or activities); 20) Completing assigned responsibilities (finishing tasks, punctuality, attendance, cooperating with teachers or caregivers, turning in homework, tidying personal areas)

Items from the Child and Adolescent Scale of Participation (CASP) are reproduced in shortened form with permission. The original instrument was developed by Bedell (2009) [26], and the German version used in this study was translated and validated by De Bock (2019) [25]. Higher scores indicate greater participation

CASP responses are rated on a 4-point scale ranging from 4 “age expected” (i.e., full participation) and 3 “somewhat limited” to 2 “very limited” and 1 “unable to participate”. An additional fifth response category of “not applicable” is provided for all items [24–26]. This option can be selected when an activity is not appropriate for the child’s age; such items are not taken into account in the calculation of the final score, according to the existing CASP guidelines published by Bedell et al. 2004 and 2009 [26, 44]. The use of ‘not applicable’ responses is part of the recommended scoring procedure and does not in itself invalidate score calculation [26, 44]. Total and subsection summary scores are calculated by summing all applicable item ratings, dividing by the maximum possible score for the applicable items (i.e., number of applicable items × 4; if all items are applicable, total maximum = 80), and multiplying by 100 to yield a 0–100 scale, with higher scores indicating greater participation [45]. The instrument also offers three open-ended questions that can be used for individualized family-centred care planning. It was shown to have a good to excellent content validity, excellent internal consistency, and good to excellent test-retest reliability [25]. Only children without missing data on the CASP as the primary dependent variable were included into the analysis.

Independent variables were chosen based on the biopsychosocial model and the ICF framework, as follows:

### Environmental factors

- Parental educational attainment, assessed according to the International Standard Classification of Education (ISCED) [46], we considered the highest educational attainment grouping the vocational and school education (low, medium, or high education level) taking into account what the parent who was answering the questionnaire reported about both parents. The highest educational attainment (medium and high categories) was the reference category vs. low educational attainment.
- Parental native language (German native language vs. non-native German language). With German native language being the reference category. We do not have detailed information about the languages spoken.

### Factors related to health and functioning

- Children’s reported reason for referral to the SPCs, i.e., physical impairment, cognitive impairment, both and other reasons for appointment in SPCs. This information became available through the SPCs documentation and the questionnaire. For regression analyses, the variable was categorized to assess impairment complexity, i.e., through dichotomization into children with physical or cognitive impairment only (reference category) versus children with both physical and cognitive impairments (higher complexity), while we also kept the category of other reasons for referral to the SPCs.
- HRQoL of CSHCN was assessed using the report short form of Disabkids [47–49], a 12-item questionnaire for children and adolescents with chronic health conditions. The instrument provides a global score that summarises the children’s overall HRQoL; minimum and maximum possible values (0–100), the higher the score, the better the HRQoL. It has solid psychometric characteristics and offers normative values of children with various chronic health conditions. Disabkids includes two formats: one that is filled in by the child (eight years or older), HRQoL - child version, and another by the parent as a proxy for the child, HRQoL - parent version. As only 203 children (36.80%, less than half of the sample) were eligible to answer the questionnaire and have completed the questionnaire, we based our study and analysis on the HRQoL parent version, which was completed by 465 parents (84.40%) [47–49].

The covariates in the regression models were child age, classified in age groups (3–6; 7–10; 11–18 years old, according to the German school system), and child’s sex (male; female); for the regression analysis, female sex and age group 3 to 6 were reference categories. For descriptive analysis, age was used in the most detailed categories available in our data set (3–4, 5–6, 7–10, 11–14, 15–18 years).

### Data analysis

Firstly, descriptive analyses were performed to describe the distribution of the outcome social participation of children as well as of the socio-demographic characteristics and other potential correlates/covariates within the sample, including missing data. Metric variables were expressed as means (M) with standard deviations (± SD), and categorical variables were expressed as absolute (n) and relative frequencies (%).

Secondly, we tested the normality and distribution of all variables using Shapiro-Wilk test, and histograms. Group comparisons were performed using Mann–Whitney U tests for two-group comparisons and Kruskal–Wallis tests for comparisons across age groups. Multi-comparison tests and analyses of the models’ covariance structure were performed by applying correlation tests and building a correlation matrix. The cut-off point for multicollinearity and stronger correlation was considered 0.70 or higher [50, 51]. Univariable linear regressions introduced all dependent variables pairwise (total CASP scale, home subscale, community subscale, school subscale and living activities subscale) and all independent variables separately and adjusted by age and sex.

Next, five multivariable models using linear regression were performed as a complete case analysis, considering *p* < 0.05 as significance, and *p* < 0.1 as trends to significance levels: (Model 1) total CASP scale, (Model 2) home subscale, (Model 3) community subscale, (Model 4) school subscale and (Model 5) living activities subscale. Regression linear coefficients (β) were reported with 95% confidence intervals (95% CI). Positive β values indicate a directly proportional association, and negative β values indicate an inversely proportional association. Variance Inflation Factor (VIF) and R-squared were analysed to evaluate fit parameters.

All analyses were performed using Stata 16.0 (Stata Corp., College Station, TX, USA) [52].

## Results

### Participants

A total of 628 children were recruited. After excluding those aged younger than 3 years (*n* = 8; 1.3%) and thus not eligible to be reported on via CASP, and those with missing CASP total score and subscales (*n* = 69; 10.99%), 551 participants remained for descriptive analyses (Table 2). Children were predominantly female (68.24%); 25.59% aged five to six and 34.66% from seven to ten years old (Table 2). All of them were registered in an SPCs to undergo care according to their impairment (17.24% physical impairment, 16.88% cognitive impairment, 33.03% physical and cognitive impairment, and 32.67% had other reasons for appointments in the SPCs). Most of the parents giving the proxy report were mothers (84.94%), and the majority of families and included CSHCN had a low educational attainment (35.75%). The mean score of HRQoL was 63.74 ± 23.77; range: 0–100.

**Table 2:** Characteristics of 551 families and children with special healthcare needs, Germany, from 11/2018 to 01/2020

Sample Characteristics	Categories	*n* (%) [mean (SD)]	Missing values
Family characteristics			
Age (years)	18–34	127 (23.05)	8 (1.45)
	≥ 35	416 (75.50)	
Sex	Female	493 (89.47)	16 (2.90)
	Male	42 (7.62)	
Caregiver	Mother	468 (84.94)	6 (1.09)
	Father	34 (6.17)	
	Others	43 (7.80)	
Parental educational attainment	Low	197 (35.75)	129 (23.41)
	Medium	63 (11.43)	
	High	162 (29.40)	
German native language	German native language	473 (85.84)	10 (1.81)
	Non-native German language	68 (12.34)	
Children characteristics			
HRQoL		(63.74 [23.77])	86 (15.61)
Age (years)	3–4	104 (18.87)	6 (1.09)
	5–6	141 (25.59)	
	7–10	191 (34.66)	
	11–14	95 (17.24)	
	15–18	14 (2.54)	
Age categories (as introduced into regression analysis)	3–6 (Kindergarten)	245 (44.46)	
	7–10 (Elementary school)	191 (34.66)	
	11–18	109 (19.78)	
Sex	Female	376 (68.24)	10 (1.81)
	Male	165 (29.95)	
Reported reason for referral to the SPCs	Physical impairment	93 (16.88)	9 (1.63)
	Cognitive impairment	182 (33.03)	
	Physical and cognitive impairment	180 (32.67)	
	Other reason for appointment	207 (33.6)	
Social participation			
Social participation	Total score of CASP	[75.99 (20.03)]	0 (0.00)
	Home subscale	[79.09 (20.18)]	0 (0.00)
	Community subscale	[75.82 (22.79)]	0 (0.00)
	School subscale	[79.44 (18.71)]	0 (0.00)
	Living Activities subscale	[68.07 (24.81)]	0 (0.00)

*CASP* Child and adolescent scale of social participation of children (0 from 100 scale, the higher is the score, the better is the social participation, *HRQoL* health-related quality of life, continuous variables are expressed as means with standard deviations (SD), categorical variables are expressed as absolute (*n*) and relative frequencies (%); SPCs, Social Paediatric Centres

^a^the highest educational attainment was assessed by the International Standard Classification of Education (ISCED) [46], one parent filled out the item for both parents, and we took into account the highest level from both parents

^b^ Other’ reasons refer to children without an established diagnosis at the time of consultation and/or whose condition was still under investigation. Reported reasons for referral to the SPCs were not mutually exclusive. Parents could indicate more than one reason; therefore, frequencies and percentages across categories do not sum to the total sample size

Regarding social participation, the mean CASP total score was 75.99 ± 20.03 (range: 25–100). Among the subscales, mean scores were highest in the school area (79.44 ± 18.71; range: 25–100) and lowest in the living activities area (68.07 ± 24.81; range: 25–100).

The distribution of item-level CASP responses in the total sample is presented in Supplementary Table S1. Full participation was highest for communication at home (60.98%), use of learning materials (56.99%), activities with friends in the neighbourhood (53.72%), and communication at school (52.81%). The lowest levels of participation were found in the living activities items, mostly in the use of transportation (17.24%), planning daily routine (28.68%), and responsibility for daily activities (28.68%).

The distribution of item-level CASP responses by child age group is shown in Supplementary Table S2. Younger children more often had “not applicable” responses in living activities items, particularly planning daily routine in children aged 3–4 years (51.92%) and 5–6 years (32.62%), use of transportation in children aged 3–4 years (41.35%) and 5–6 years (24.82%), and responsibility for daily activities in children aged 3–4 years (52.88%) and 5–6 years (43.97%). The mean, minimum and maximum CASP scores according to the independent variables categories are shown in supplementary material, Table S3. CASP total and subscales mean scores increased with age (CASP total score: 74.91 in children aged 3–6 years; 76.63 in those aged 7–10 years; 77.00 in those aged 11–18 years). Mean CASP total scores were 75.73 in children with low parental educational attainment compared to 76.08 in those with medium and high educational attainment, and 76.03 in children with German native language compared to 75.72 in those with non-native German language. Also, it was higher in male (76.20) than in female (75.69).

### Social participation of children with special healthcare needs and correlates

Regarding the multivariate analysis, the preliminary tests and matrix correlation indicated no sign of multicollinearity (Table S4, supplementary material). All Spearman correlation coefficients between independent variables remained below the multicollinearity threshold of 0.70. Because the regression analyses were based on complete-case data, the analytic sample was reduced to 350 children and families. The characteristics of this complete-case sample are presented in Supplementary Table S5. Also, the univariable linear regressions with all dependent variables and independent variables, are available in supplementary material, Table S6.

### Regression on total score CASP

The total CASP score (Model 1) was associated with age (7 to 10 years: β = 8.06; 11 to 18 years: β = 10.80), showing significantly higher coefficients for older age groups compared to the reference group (3 to 6 years old) (Table 3). HRQoL was also positively associated with total CASP scores (β = 0.42). Children with both types of impairments, physical and cognitive, had lower CASP scores (β = -4.33), compared to those with only a physical or a cognitive impairment (Table 3). Non-native German language was associated with lower participation in the total CASP score and in the subscales, school and living activities (*p* = 0.09, 0.09, and 0.08, respectively).

**Table 3:** Correlates associated with social participation of children with special healthcare needs in Germany as reported by their parent caregivers between 11/2018 and 01/2020

Variables	β	CI [lower limit; upper limit]	*p*	Mean VIF	*R* ^2^
Total CASP scale (*n* = 350)					
Parental educational attainment – Medium and high	Ref			1.12	0.27
Parental educational attainment – Low	-2.02	[-5.62; 1.57]	0.26		
German native language	Ref				
Non-native German language	-4.87	[-10.64; 0.90]	0.09		
Female	Ref				
Male	2.43	[-1.67; 6.54]	0.24		
Physical or cognitive impairment	Ref				
Both impairments and others	-4.33	[-7.81; -0.84]	0.01		
HRQoL (parent)	0.42	[0.34; 0.49]	< 0.01		
Age − 3 to 6 years old	Ref				
Age − 7 to 10 years old	8.06	[4.15; 11.98]	< 0.01		
Age − 11 to 18 years old	10.80	[5.97; 15.62]	< 0.01		
CASP subscales					
Home (*n* = 350)					
Parental educational attainment – Medium/high	Ref			1.12	0.24
Low	-0.87	[-4.61; 2.85]	0.64		
German native language	Ref				
Non-native German	-3.99	[-10.29; 2.30]	0.21		
Female	Ref				
Male	2.27	[-1.96; 6.51]	0.29		
Physical/cognitive impairment	Ref				
Both impairments and others	-4.07	[-7.71; -0.44]	0.02		
HRQoL (parent)	0.41	[0.33; 0.49]	< 0.01		
Age − 3 to 6 years old	Ref				
Age − 7 to 10 years old	8.67	[4.59; 12.75]	< 0.01		
Age − 11 to 18 years old	9.77	[4.68; 14.85]	< 0.01		
Community (*n* = 350)					
Parental educational attainment – Medium/high	Ref			1.12	0.24
Low	-1.59	[-5.82; 2.64]	0.46		
German native language	Ref				
Non-native German	-5.04	[-12.10; 2.01]	0.16		
Female	Ref				
Male	4.13	[-0.58; 8.85]	0.08		
Physical/cognitive impairment	Ref				
Both impairments and others	-4.18	[-8.35; -0.00]	0.05		
HRQoL (parent)	0.46	[0.37; 0.55]	< 0.01		
Age − 3 to 6 years old	Ref				
Age − 7 to 10 years old	9.37	[4.68; 14.07]	< 0.01		
Age − 11 to 18 years old	13.59	[8.11; 19.06]	< 0.01		
School (*n* = 350)					
Parental educational attainment – Medium/high	Ref			1.12	0.26
Low	-0.53	[-3.86; 2.79]	0.75		
German native language	Ref				
Non-native German	-4.62	[-10.02; 0.77]	0.09		
Female	Ref				
Male	2.14	[-1.66; 5.96]	0.26		
Physical/cognitive impairment	Ref				
Both impairments and others	-4.60	[-7.82; -1.38]	0.00		
HRQoL (parent)	0.36	[0.29; 0.43]	< 0.01		
Age − 3 to 6 years old	Ref				
Age − 7 to 10 years old	9.89	[6.28; 13.50]	< 0.01		
Age − 11 to 18 years old	11.90	[7.44; 16.35]	< 0.01		
Living Activities (*n* = 350)					
Parental educational attainment – Medium/high	Ref			1.12	0.20
Low	-5.71	[-10.36; -1.05]	0.01		
German native language	Ref				
Non-native German	-6.48	[-13.84; 0.88]	0.08		
Female	Ref				
Male	1.80	[-3.43; 7.04]	0.49		
Physical/cognitive impairment	Ref				
Both impairments and others	-3.90	[-8.59; 0.77]	0.10		
HRQoL (parent)	0.45	[0.36; 0.54]	< 0.01		
Age − 3 to 6 years old	Ref				
Age − 7 to 10 years old	4.50	[-0.69; 9.69]	0.08		
Age − 11 to 18 years old	9.54	[3.17; 15.91]	0.00		

Multivariable linear regression models: (1) total CASP scale, (2) home subscale, (3) community subscale, (4) school subscale and (5) living activities subscale to identify which correlates are associated with social participation. Ref, reference category, *CASP* Child and Adolescent Scale of Participation, *HRQoL* children’s Health-related quality of life, β Standardized Regression Coefficient, *VIF* Variance Inflation Factor, R-squared, coefficient of determination

### Regression on CASP subscales

The models assessing the home (Model 2), community (Model 3), and school subscales (Model 4) showed similar patterns (Table 3). Older age groups, 7 to 10 and 11 to 18 years, were positively associated with higher scores in each subscale (Home: β = 8.67 and 9.77; Community: β = 9.37 and 13.59; School: β = 9.89 and 11.90) and higher HRQoL as well (Home: β = 0.41; Community: β = 0.46; School: β = 0.36). By contrast, children exhibiting both types of impairments, physical and cognitive, had consistently lower CASP scores (Home: β = -4.07; School: β = -4.60).

The items belonging to living activities (Model 5) rely more on autonomy, executive functions, and responsibility (planning, time management, money skills) than those of other, as previously mentioned in Table 1. Older age (11 to 18 years) was associated with higher scores in CASP (β = 9.54), as shown in Table 3. In this subscale, more correlates were found. This model showed that low educational attainment (versus medium and high (β = -5.71)) was negatively associated with score in CASP living activities subscale. Regarding age and HRQoL, the pattern continued to be similar with the previously models, and HRQoL remained a significant positive correlator (β = 0.45).

Male sex has a trend to be associated with higher participation in the community domain (*p* = 0.08), and children aged 7–10 years showed higher scores in the living activities subscale (*p* = 0.08). Impairment complexity was borderline significantly associated with lower participation in the community model (*p* = 0.05) and showed a similar trend in living activities (*p* = 0.10).

## Discussion

This study provides a comprehensive multi-site assessment of correlates of social participation of CSHCN in routine care with different diagnoses and conditions treated in SPCs in Germany. The findings highlight that child age and HRQoL are consistently positively related to social participation across domains, while low parental educational attainment was related to lower values in the living activities subscale. It supports the assumption that participation is not determined by diagnosis alone, but emerges from the dynamic interplay between individual and environmental conditions within the biopsychosocial framework of the ICF.

The main correlates differ across the CASP subscales (home, community, school, and living activity): child age was positively related to all subscales; the complexity of impairment operationalized based upon the primary clinical reason for referral to the SPCs was negatively associated with home, and school participation, but not with the community and living activities subscale. Only the living activities subscale showed associations with family characteristics, and was lower with lower parental educational attainment. When also acknowledging variables with a trend to significance, other variables in other domains, such as non-native German language, was associated with a lower total score. This might be explained by the fact that non-German language was previously found associated with lower healthy literacy [53], access to the healthcare system [54], communication with institutions, and thus also potentially lower participation opportunities [54]. This finding is in line with previous studies conducted in typically developing children from other socioeconomically disadvantaged populations [55], but also in CSCHN, in which ethnic minority status, limited material and social resources and low parental education correlated with social participation [31]. These findings are consistent with previous studies showing that participation in children and youths with disabilities is shaped by multiple child- and family-related factors. A study conducted in Turkey observed that participation in leisure activities among children with special needs was associated with age, gender, and socioeconomic status, while also emphasizing the importance of family guidance and environmental support for participation [56]. Similarly, a Swedish study investigating participation and independence in children and youths with disabilities found that comprehension difficulties, speech abilities, pain, age, seizures, and motor functioning were important predictors of participation and independence in daily activities [57].

As our sample was recruited across multiple sites as CSHCN in routine care of German SPCs, the sample may differ from community-based or diagnosis-specific samples examined in previous studies. In this line, our sample had a high percentage of low-educational background, which enhances its relevance when interested in how to increase social participation in groups with multiple social and health-related vulnerabilities.

In addition, our study analysed CASP domains separately besides the total score, which can reveal domain-specific correlates which other studies using more global participation measures could not show. Although we did not measure income or socioeconomic status that could represent material deprivation, out results on the relevance of low parental education as a main determinants of social participation in at least one domain is comparable to a large population-based study from Germany, which showed that individuals exposed to adverse childhood experiences had lower perceived social participation and lower HRQoL than non-exposed individuals, with the strongest disadvantage observed in those with polyadversity [58].

Moreover, concepts with overlapping dimensions such as HRQoL, and impairment complexity also significantly correlate with social participation in CSHCN in our study. Both concepts are linked to participation, as impairments may restrict activities and opportunities, and HRQoL reflects how health conditions affect everyday life, perceived physical, emotional, and social functioning. Therefore, promoting social participation, as also suggested by the ICF, requires interventions that address both the individual needs of the child and the resources and support available within the family environment together. In addition, we show that HRQoL is a concept inherently related to social participation, which explains the great correlation of both, corroborating also with previous studies [25, 59]. Our study adds to the literature by examining family characteristics in relation to social participation among CSHCN and by analysing participation across distinct domains, including home, community, school, and daily living activities [60, 61].

Three main transferable lessons emerge from our findings. First that social participation is not determined by health conditions alone, but, as previously said, is also dependent on environmental factors such as parental education. This may reflect challenges that are common across countries, not only Germany, but also in low- and middle-income countries and in contexts affected by war or displacement. Our findings therefore suggest that addressing family-level and broader environmental barriers is essential across different countries for successfully increasing social participation of children with chronic conditions and disabilities from a public health perspective. It actually is also a matter of social policy and child rights protection.

Second, social participation is a dynamic construct that develops across the life course and is experienced differently at different developmental stages. From a practice point of view, early age seems to be a vulnerable phase for social participation, as children are not yet routinely reached through compulsory schooling. Also, CSHCN may have more challenges to access the educational and health system [62, 63]. Thus, tailored education by age, illness, and family needs prioritising individualised content, multimodal delivery and flexible approaches are key [62], and may be important specifically for younger children and CSHCN.

Third, we observed that participation varies across domains (home, community, school, and daily living activities), suggesting that interventions might have to be domain-specific and tailored to the child’s everyday environments and participation preferences. Assessing participation preferences is a key element of the PARTCHILD intervention [33], a program to improve participation-centred care for CSHCN. It helps healthcare teams identify children’s and parents’ preferences, set participation goals, and support shared decision-making involving different stakeholders of the care network. The program includes staff training, a documentation software, and tailored support to bring these ideas into routine care [33]. Individual participation preferences traditionally also have been assessed in care by the Canadian Occupational Performance Measure (COPM) [64, 65] and could serve as a starting point for care directed at improving social participation in preferred subdomains. In the future, social participation measures such as the CASP and Children’s Assessment of Participation and Enjoyment [66] should therefore be complemented by participation preferences measurement, for which adequate instruments hitherto are still lacking.

In the next sections, we will discuss the specific correlations of social participation within our models.

### Social participation of children with special healthcare needs and its correlates

#### Age (7–10 and 11–18 years) and social participation

Older children (ages categories: from 7 to 10 and from 11 to 18 years) showed consistently higher CASP scores across all domains, community, school, home and living activities subscales, compared with younger children (3–6 years). In addition, children aged 7–10 years showed higher scores in the living activities subscale, with a trend toward statistical significance (*p* = 0.08). This pattern may reflect the greater opportunities for social participation that come with age, as children develop more advanced skills, for instance, sports, language, communication, and socio-cognitive skills [67]. However, the opposite direction of correlation is also possible: children that seek developmental care earlier and at a younger age, e.g., with difficulties in language skills, may generally have a lower social participation and more severe symptoms than those only seeking care at an older age (bias by indication). A recent study demonstrated that parents of children with developmental language disorders reported aggressive behaviour, and social difficulties during the early years of primary school due to language impairments [68]. These challenges can lead to difficulties in social participation. Therefore, more longitudinal research is needed to identify the personal, communicative and environmental factors that may interact with participation outcomes [69]. It would be interesting for future studies to compare CASP-based findings with those obtained using instruments designed specifically for younger children, for instance, the Young Children’s Participation and Environment Measure, which focuses on children aged 0–5 years [70, 71] to further investigate the interaction between age and participation.

### Parental German native language, parental educational attainment and social participation

In our study, children whose parents were native German speakers showed higher social participation in all domains (Table S2, supplementary material). However, this variable was not significantly associated with social participation, although it showed trends toward statistical significance in the total CASP score and in the school and living activities subscales (*p* = 0.09, 0.09, and 0.08, respectively). More studies with a larger sample size would be interesting to verify the association between parental native language and social participation, as we lost power through missing data reducing the complete case sample available for regression analyses.

Parental native language may play an important role in the families’ integration into the social and cultural environment, which makes it important to specifically support social participation in CSHCN with reduced parental language skills and health literacy. Since the living activities subscale is related to planning, time management, and money skills, besides of age, the social integration of the family as well as their systems knowledge (including health literacy) might be important to eliminate potential systemic barriers to access facilities and participate. Moreover, it may have dual directions being a cycle because the presence of a CSHCN may also affect the social participation of family members, through increased caregiving demands and reduced time and resources for social activities [72]. As previously mentioned, from an ICF perspective, body functions and structures, activities, participation, and contextual factors (environmental and personal factors) are interactive and bidirectional [42]. Therefore, family resources and the social context can influence on participation outcomes, while the child’s support needs can also affect family resources over time. Our variable parental non-native German language on the one hand may not be homogenous, e.g. it may mix different languages and cultures into on non-native Germany category, which may limit its validity, and at the same time, the variable may overlap with other sociodemographic and contextual factors, such as socioeconomic status and neighbourhood deprivation, which we did not fully measure.

Children with less engaged or linguistically isolated families may face compounding challenges, leading to lower participation scores [73]. A recent study, published in 2025, on parental school involvement showed that parents who do not speak the host country language and face language difficulties or do not know how to participate in school activities due to language barriers are more likely to belong to “low involvement” or “medium personal barriers” groups, characterised by reduced engagement in school activities [74]. This parental reduced engagement in school activities may create a cumulative disadvantage for children. A large sample data analysis from Brazil indicated that higher parental involvement in children’s school is associated with better reading and math achievement in primary school, partly through increased homework completion and everyday learning opportunities [75]. Although these studies were conducted in typically developing children, they support the assumption that when parents are better integrated into key institutions, such as schools and can participate despite linguistic or structural barriers, children gain more opportunities to practise age-appropriate skills and to have better performance also in other social participation domains, for instance, in living activities (planning, time management, and money skills) [75].

More children in the low parental educational attainment category had significantly reduced participation in the living activities subscale. This subscale covers tasks that rely on planning, independence, and executive functions, such as daily responsibilities, transport, and time management [25, 26]. Low educational attainment may contribute to low health literacy. It means difficulties in recognizing a need for support, evaluating available offers, and engaging in health-related services [76]. Individuals with disadvantageous health determinants are more likely to report inadequate health literacy, particularly with regard to support child development [77]. A systematic review showed that relevant support mechanisms may include individually tailored programmes, such as individualized coaching, group mentoring, and a five-step individualized approach involving the establishment of a collaborative relationship with parents, setting mutually agreed goals, selecting the most appropriate therapeutic activity for achieving the goal, and supporting parents [78]. Group-based approaches aimed at enhancing interactions between participants, with reported increases in positive interactions and decreased solitary play, may also be relevant for supporting social participation [78]. In addition, supporting children’s decision-making, social support, and autonomy in play, including honouring personal preferences and allowing children to self-select active play activities, may be important [79]. In terms of physical structure, adapted activities, assistive technologies, inclusive playground designs, and access to community-based programs also are effectives strategies to support social participation beyond the child alone, but including environmental, social, and structural conditions [79]. In Germany (e.g., “Strong parents – strong children” [80]), and in England (e.g., the Triple P-Positive Parenting Program in England [81]), there are parent education programs that aim to support families in raising children. These programmes are mainly universal rather than targeted [82], such as for CSHCN or parents with low educational attainment, but instead address child development, family phases, everyday family tasks, different family constellations, family circumstances [83] and stressful situations [84]. These topics may support the family to raise the children. If adapted to the needs of CSHCN and families with lower educational attainment, such programmes and their infrastructures could more specifically address participation-related skills and barriers. Parental education support could be a tool for delivering specific knowledge on social participation and integration for these families, especially in case of low parental educational attainment. Greater integration of the parents into the community and support, for instance, the previously mentioned parent education programs or social prescribing [85], may also improve social participation for the whole family. Social prescribing or facilitated access to local activities, may support families in connecting with resources and participation opportunities. The influence of parent education programs or social prescribing as a way to promote social participation of CSHCN and their families, however, has not been studied extensively.

### Health-related quality of life, impairment complexity and social participation

In our sample, the HRQoL mean was 63.74 ± 23.77, which is substantially lower than values reported in the German validation study of the KINDLR instrument (24-items questionnaire also developed to measure HRQoL [86, 87]), where HRQoL was 73.77 ± 9.81 in a neuropediatric outpatient sample, 71.31 ± 13.48 in a paediatric rehabilitation sample, 83.30 ± 8.02 in a population-based sample, and 79.99 ± 10.24 in the total sample [25]. The lower HRQoL observed in our study may reflect a lower HRQoL in children with combined physical and cognitive impairments, greater functional limitations, and higher complexity of care needs in the SPCs setting compared to the mixed clinical and community samples cited. In our study, HRQoL emerged also as a consistent positive cross-sectional correlate across all CASP models. Children with higher HRQoL showed significantly greater participation in home, community, school and living activities. This correlation is not surprising, as there exists a great overlap between both concepts [25]. Compared to quality of life, HRQoL focuses on the impact of health (including physical, mental, and social functioning) and healthcare interventions on a person’s ability to live a fulfilling life, however does not encompass non-health domains such as financial security [88]. Many times, studies showed no big difference between samples of children with and without chronic disease [89], maybe explained by the fact that expectations of quality of life differ greatly between those without chronic disease and those with, with generally lower expectations in those with pre-existing disease. In contrast, social participation, as measured by the CASP, captures a child’s actual involvement in everyday life across home, school, community, and daily living, and is influenced not only by health status but also by environmental, social, and personal factors. Participation estimates are consistently lower in children with a diagnosis. For example, in our previous study in Germany, children without chronic conditions had a mean CASP score of 98.2. Social participation was approximately 3.4% lower in children with mild chronic conditions and 28.7% lower in those with severe chronic conditions, while there was a significant correlation between participation and HRQoL [25]. Despite this, participation should not be substituted by HRQoL [25], but a situation might be much more relevantly assessed when the measures are combined. For instance, if the children are not feeling well and have a low HRQoL, often due to physical limitations, pain, emotional distress, or disability, consequently they may experience greater barriers to engaging in social, occupational, and community activities, as demonstrated in children from 8 to 18 years old [90].

Another correlate found in our results was the impairment complexity. CSHCN with both physical and cognitive impairment demonstrated lower social participation in comparison with CSHCN with physical or cognitive impairment. Thus, these results suggest that greater severity of disability and complexity of impairment and the presence of environmental barriers are associated with lower levels of social participation. Better physical, emotional, and social functioning, may enable greater participation in social activities. As we did not include a control group of typically developing children, the analytical contrast in this study was between single impairment occurrence and multiple impairment occurrence. However, previous study using the CASP has shown that children with mild disease had higher participation than those with severe disease, and similar levels of participation as children without chronic health conditions [25]. Future studies should investigate other conditions, including psychosocial disorders such as anxiety, in order to better differentiate the role of condition type, co-occurrence, and severity in social participation.

In this context, based on our result showing an association between social participation and HRQoL, we can assume that interventions aimed at improving HRQoL may also increase social participation. For this reason, the American Academy of Pediatrics recommends to support CSHCN and their parents using a family-cantered approach focusing on function, family, fitness, fun, friends, and future [91, 92]. In SPCs, where our data were collected, CSHCN are already receiving interdisciplinary treatment based on this approach [93], which may act as a protective factor, mitigating some negative effects of low socioeconomic status or complex health conditions. However, it may be important that SPCs also make partnerships with the local communities and offer adaptive recreation and participation in community or school-based adapted physical education, therapeutic recreation, and adaptive sports [94]. Such partnerships can create more opportunities for children to engage in everyday life activities, for example, at school, home, community and living, potentially enhancing their social participation. However, studies on the effectiveness of these strategies are lacking despite their potential of improving the CSHCN development. Our findings highlight those correlates, such as parental education and language background, influence participation, suggesting that connecting families to community resources could help reduce participation gaps.

### Limitations and strengths

This study’s strength is that it sheds light on a construct that only a limited number of existing studies focus on, and on a specifically vulnerable child population with an assumable broad range of diagnoses due to very broad inclusion criteria as compared to many disease-specific studies on social participation of CSHCN so far. While SPCs are a key healthcare area of CSHCN in Germany, recruitment via SPCs might not be fully representative for CSHCN in Germany, given that SPCs offer treatment for 466.00 children per year, i.e. approximately 3.5% of all children in Germany [34]. However, SPC represent a real routine care environment, and our study encompasses a sample recruited across multiple (*n* = 15) sites distributed across all parts of Germany, which again increases representativeness. By focusing on an SPCs-based population, the study however captures a segment of CSHCN with mostly complex needs that may benefit most from targeted and systematized interventions, underscoring the value of SPCs not only in delivering care but also in screening, tracking and supporting social outcomes. There is currently no evidence directly comparing social participation outcomes between CSHCN who receive care in SPCs and those who do not. To our knowledge, this is the first study to examine social participation in SPCs sample in Germany. However, in line with previous reviews [31, 95], participation is a complex construct that is associated with social factors. As these reviews did not differentiate participation by treatment attendance [31, 95], we expect that CSHCN not receiving treatment at SPCs may experience similar or lower levels of participation if exposed to comparable disadvantages.

Notably, this sample included a high proportion of families with low educational attainment, a vulnerable group often underrepresented in research. This enhances the equity and potential relevance of the findings. However, the regression analyses were conducted using complete-case analysis, reducing the sample size analysed in regression models. This reduction may affect statistical power and representativeness, but retains unbiased estimates as compared to multiple imputation. In the complete-case sample, the proportion of families with low parental educational attainment further increased from 35.75% in the full sample to 46.28%, which is relatively high and may exceed proportions reported in other studies. In addition, some shifts in sample composition were observed, including a higher proportion of male children (from 29.95% to 69.71%) and a slight increase in younger age groups (3–6 years: from 44.46%.

to 45.14%), while HRQoL (from 63.74 to 64.16) and parental language distribution (from 12.34% to 12.85% German native language speakers) remained comparable (Table S5, supplementary material). These changes should be considered when interpreting the regression results and – in terms of high percentage of low education families - increases relevance of findings.

One inclusion criterion required was the ability to complete the questionnaire in German, which may have excluded families with very limited language proficiency and thus may partly underrepresent groups with immigration background. In 2023, approximately 17% of German households speak a language other than German at home [96, 97], and in our complete-case sample, we had 45 participants (12.85%) that reported a non-native German language. Another limitation is how impairment and disease severity were operationalized: They fully relied on the reported reason for referral to the SPCs variable, via parent reports and SPCs records, rather than standardized clinical assessments, which may introduce misclassification or variability across sites. In addition, we did not measure socioeconomic status fully, i.e., household income was not included. A full assessment of socioeconomic status could have provided a more precise understanding of socioeconomic influences beyond parental education and language background. Also, due to the cross-sectional study design, no causal conclusions can be drawn, and the possibility of bidirectional associations must be considered. Regarding the families of non-participants, we also did not systematically document the reasons why they declined to complete the questionnaire. Therefore, we cannot compare decliners and participants in terms of sociodemographic or clinical characteristics.

## Conclusions

This study offers important insights into the correlates of social participation among CSHCN in Germany. The results demonstrate that social participation is not only determined by individual characteristics related to health and functioning, such as HRQoL and impairment complexity, but also, in the living activities domain (planning, time management, money skills), by key environmental, social factors, such as parental education and, to a lesser extent, native language. These findings point to a compounded vulnerability, where biological, functional, and social disadvantages converge to reduce social participation and may reinforce one another. Future research and practice should therefore focus on targeted strategies for socially vulnerable groups, including individually tailored support such as goal-oriented guidance for families, group-based approaches that strengthen positive interactions, and support for children’s autonomy and decision-making in everyday activities. In addition, adapted activities, assistive technologies, inclusive playground designs, and improved access to community-based programs may help address environmental barriers to participation. SPCs could strengthen collaboration with schools, sports clubs, and cultural organisations to expand accessible opportunities, for instance, by co-creation projects, or workshops with families’ associations. Building stronger connections between families and community services may help reduce participation disparities and improve developmental outcomes for CSHCN. In addition, it is recommended to adopt longitudinal and mixed-methods approaches to better understand causal pathways and to capture the dynamics of social participation over time.

## Supplementary Information

Supplementary Material 1.

## Data Availability

The original contributions presented in the study are included in the article, further inquiries can be directed to the corresponding author/s.

## Abbreviations

CSHCN: Children with special healthcare needs
CASP: Child and Adolescent Scale of Participation
HRQoL: children’s Health-related quality of life
ICF: International Classification of Functioning, Disability and Health
SPCs: Social Paediatric Centres
SD: standard deviations
VIF: Variance Inflation Factor
M: means
95% CI: 95% confidence intervals
β: 95% confidence intervals
