# Supplementary material for: Correlates of social participation of children with special healthcare needs: baseline of the PART-CHILD study
Source: BMC Pediatr. 2026 Jun 29;26:600. doi: 10.1186/s12887-026-07233-3 (PMC13321687; doi:10.1186/s12887-026-07233-3)
Supplement: Supplementary file 1 — Supplementary Material 1. [file 12887_2026_7233_MOESM1_ESM.docx]

**Supplementary material**

Table S1. Distribution of item-level responses on the Child and Adolescent Scale of Participation (CASP) in the total sample (n = 551)

| **Items (CASP)**  **n (%)** | **Full participation** | **Somewhat limited** | **Very limited** | **Unable** | **Not applicable** | **Missing** | **Total** |
| --- | --- | --- | --- | --- | --- | --- | --- |
|  | ***Home participation*** | | | | | | |
| 1. Social activities with family at home | 184 (33.39) | 217 (39.38) | 123 (22.32) | 20 (3.63) | 0 (0.00) | 7 (1.27) | 551 |
| 2. Social activities with friends at home | 240 (43.56) | 184 (33.39) | 94 (17.06) | 32 (5.81) | 1 (0.18) | 0 (0.00) | 551 |
| 3. Participation in household responsibilities | 217 (39.38) | 167 (30.31) | 111 (20.15) | 45 (8.17) | 11 (2.00) | 0 (0.00) | 551 |
| 4. Self-care (eating, dressing, hygiene) | 247 (44.83) | 166 (30.13) | 93 (16.88) | 42 (7.62) | 3 (0.54) | 0 (0.00) | 551 |
| 5. Mobility at home | 245 (44.46) | 138 (25.05) | 99 (17.97) | 67 (12.16) | 2 (0.36) | 0 (0.00) | 551 |
| 6. Communication at home | 336 (60.98) | 107 (19.42) | 69 (12.52) | 35 (6.35) | 4 (0.73) | 0 (0.00) | 551 |
|  | ***Community participation*** | | | | | | |
| 7. Activities with friends in neighborhood | 296 (53.72) | 151 (27.40) | 76 (13.79) | 24 (4.36) | 4 (0.73) | 0 (0.00) | 551 |
| 8. Organized community activities | 215 (39.02) | 147 (26.68) | 132 (23.96) | 52 (9.44) | 5 (0.91) | 0 (0.00) | 551 |
| 9. Mobility in community | 187 (33.94) | 148 (26.86) | 124 (22.50) | 68 (12.34) | 24 (4.36) | 0 (0.00) | 551 |
| 10. Communication in community | 283 (51.36) | 105 (19.06) | 77 (13.97) | 69 (12.52) | 17 (3.09) | 0 (0.00) | 551 |
|  | ***School participation*** | | | | | | |
| 11. Participation in classroom activities | 273 (49.55) | 145 (26.32) | 83 (15.06) | 46 (8.35) | 4 (0.73) | 0 (0.00) | 551 |
| 12. Social/leisure activities at school | 182 (33.03) | 187 (33.94) | 143 (25.95) | 38 (6.90) | 1 (0.18) | 0 (0.00) | 551 |
| 13. Mobility at school | 191 (34.66) | 213 (38.66) | 127 (23.05) | 19 (3.45) | 1 (0.18) | 0 (0.00) | 551 |
| 14. Use of learning materials | 314 (56.99) | 144 (26.13) | 70 (12.70) | 22 (3.99) | 1 (0.18) | 0 (0.00) | 551 |
| 15. Communication at school | 291 (52.81) | 155 (28.13) | 72 (13.07) | 25 (4.54) | 8 (1.45) | 0 (0.00) | 551 |
|  | ***Living activities*** | | | | | | |
| 16. Household tasks | 273 (49.55) | 167 (30.31) | 78 (14.16) | 31 (5.63) | 2 (0.36) | 0 (0.00) | 551 |
| 17. Shopping and money use | 223 (40.47) | 166 (30.13) | 97 (17.60) | 56 (10.16) | 9 (1.63) | 0 (0.00) | 551 |
| 18. Planning daily routine | 158 (28.68) | 87 (15.79) | 76 (13.79) | 115 (20.87) | 115 (20.87) | 0 (0.00) | 551 |
| 19. Use of transportation | 95 (17.24) | 131 (23.77) | 96 (17.42) | 142 (25.77) | 87 (15.79) | 0 (0.00) | 551 |
| 20. Responsibility for daily activities | 158 (28.68) | 62 (11.25) | 46 (8.35) | 131 (23.77) | 154 (27.95) | 0 (0.00) | 551 |

n, absolute frequency; %, relative frequency.

Table S2. Distribution of item-level Child and Adolescent Scale of Participation (CASP) responses by child age group.

| **Items (CASP)**  **n (%)** | **Child Age** | **Full participation** | **Somewhat limited** | **Very limited** | **Unable** | **Not applicable** | **Total** |
| --- | --- | --- | --- | --- | --- | --- | --- |
| 1. Social activities with family at home | 3–4 | 29 (27.88) | 46 (44.23) | 24 (23.08) | 3 (2.88) | 2 (1.92) | 104 |
|  | 5–6 | 50 (35.46) | 57 (40.43) | 31 (21.99) | 2 (1.42) | 1 (0.71) | 141 |
|  | 7–10 | 68 (35.60) | 70 (36.65) | 46 (24.08) | 7 (3.66) | 0 (0.00) | 191 |
|  | 11–14 | 32 (33.68) | 37 (38.95) | 18 (18.95) | 7 (7.37) | 1 (1.05) | 95 |
|  | 15–18 | 3 (21.43) | 6 (42.86) | 2 (14.29) | 1 (7.14) | 2 (14.29) | 14 |
|  | Missing | 2 (33.33) | 1 (16.67) | 2 (33.33) | 0 (0.00) | 1 (16.67) | 6 |
| 2. Social activities with friends at home | 3–4 | 42 (40.38) | 38 (36.54) | 16 (15.38) | 8 (7.69) | 0 (0.00) | 104 |
|  | 5–6 | 62 (43.97) | 44 (31.21) | 26 (18.44) | 9 (6.38) | 0 (0.00) | 141 |
|  | 7–10 | 83 (43.46) | 63 (32.98) | 37 (19.37) | 7 (3.66) | 1 (0.52) | 191 |
|  | 11–14 | 42 (44.21) | 32 (33.68) | 14 (14.74) | 7 (7.37) | 0 (0.00) | 95 |
|  | 15–18 | 8 (57.14) | 4 (28.57) | 1 (7.14) | 1 (7.14) | 0 (0.00) | 14 |
|  | Missing | 3 (50.00) | 3 (50.00) | 0 (0.00) | 0 (0.00) | 0 (0.00) | 6 |
| 3. Participation in household responsibilities | 3–4 | 36 (34.62) | 37 (35.58) | 20 (19.23) | 8 (7.69) | 3 (2.88) | 104 |
|  | 5–6 | 55 (39.01) | 43 (30.50) | 28 (19.86) | 12 (8.51) | 3 (2.13) | 141 |
|  | 7–10 | 77 (40.31) | 53 (27.75) | 44 (23.04) | 14 (7.33) | 3 (1.57) | 191 |
|  | 11–14 | 39 (41.05) | 27 (28.42) | 18 (18.95) | 9 (9.47) | 2 (2.11) | 95 |
|  | 15–18 | 7 (50.00) | 4 (28.57) | 1 (7.14) | 2 (14.29) | 0 (0.00) | 14 |
|  | Missing | 3 (50.00) | 3 (50.00) | 0 (0.00) | 0 (0.00) | 0 (0.00) | 6 |
| 4. Self-care (eating, dressing, hygiene) | 3–4 | 42 (40.38) | 35 (33.65) | 16 (15.38) | 9 (8.65) | 2 (1.92) | 104 |
|  | 5–6 | 72 (51.06) | 39 (27.66) | 19 (13.48) | 10 (7.09) | 1 (0.71) | 141 |
|  | 7–10 | 83 (43.46) | 58 (30.37) | 37 (19.37) | 13 (6.81) | 0 (0.00) | 191 |
|  | 11–14 | 38 (40.00) | 31 (32.63) | 17 (17.89) | 9 (9.47) | 0 (0.00) | 95 |
|  | 15–18 | 8 (57.14) | 1 (7.14) | 4 (28.57) | 1 (7.14) | 0 (0.00) | 14 |
|  | Missing | 4 (66.67) | 2 (33.33) | 0 (0.00) | 0 (0.00) | 0 (0.00) | 6 |
| 5. Mobility at home | 3–4 | 32 (30.77) | 34 (32.69) | 22 (21.15) | 16 (15.38) | 0 (0.00) | 104 |
|  | 5–6 | 70 (49.65) | 28 (19.86) | 26 (18.44) | 17 (12.06) | 0 (0.00) | 141 |
|  | 7–10 | 89 (46.60) | 46 (24.08) | 36 (18.85) | 18 (9.42) | 2 (1.05) | 191 |
|  | 11–14 | 43 (45.26) | 26 (27.37) | 13 (13.68) | 13 (13.68) | 0 (0.00) | 95 |
|  | 15–18 | 8 (57.14) | 3 (21.43) | 1 (7.14) | 2 (14.29) | 0 (0.00) | 14 |
|  | Missing | 3 (50.00) | 1 (16.67) | 1 (16.67) | 1 (16.67) | 0 (0.00) | 6 |
| 6. Communication at home | 3–4 | 54 (51.92) | 21 (20.19) | 20 (19.23) | 8 (7.69) | 1 (0.96) | 104 |
|  | 5–6 | 79 (56.03) | 32 (22.70) | 20 (14.18) | 8 (5.67) | 2 (1.42) | 141 |
|  | 7–10 | 127 (66.49) | 34 (17.80) | 18 (9.42) | 12 (6.28) | 0 (0.00) | 191 |
|  | 11–14 | 63 (66.32) | 16 (16.84) | 11 (11.58) | 4 (4.21) | 1 (1.05) | 95 |
|  | 15–18 | 11 (78.57) | 1 (7.14) | 0 (0.00) | 2 (14.29) | 0 (0.00) | 14 |
|  | Missing | 2 (33.33) | 3 (50.00) | 0 (0.00) | 1 (16.67) | 0 (0.00) | 6 |
| 7. Activities with friends in neighborhood | 3–4 | 39 (37.50) | 34 (32.69) | 23 (22.12) | 7 (6.73) | 1 (0.96) | 104 |
|  | 5–6 | 71 (50.35) | 45 (31.91) | 16 (11.35) | 8 (5.67) | 1 (0.71) | 141 |
|  | 7–10 | 124 (64.92) | 39 (20.42) | 23 (12.04) | 5 (2.62) | 0 (0.00) | 191 |
|  | 11–14 | 52 (54.74) | 25 (26.32) | 13 (13.68) | 3 (3.16) | 2 (2.11) | 95 |
|  | 15–18 | 6 (42.86) | 7 (50.00) | 0 (0.00) | 1 (7.14) | 0 (0.00) | 14 |
|  | Missing | 4 (66.67) | 1 (16.67) | 1 (16.67) | 0 (0.00) | 0 (0.00) | 6 |
| 8. Organized community activities | 3–4 | 29 (27.88) | 38 (36.54) | 25 (24.04) | 9 (8.65) | 3 (2.88) | 104 |
|  | 5–6 | 61 (43.26) | 33 (23.40) | 35 (24.82) | 10 (7.09) | 2 (1.42) | 141 |
|  | 7–10 | 77 (40.31) | 49 (25.65) | 45 (23.56) | 20 (10.47) | 0 (0.00) | 191 |
|  | 11–14 | 40 (42.11) | 21 (22.11) | 23 (24.21) | 11 (11.58) | 0 (0.00) | 95 |
|  | 15–18 | 5 (35.71) | 5 (35.71) | 3 (21.43) | 1 (7.14) | 0 (0.00) | 14 |
|  | Missing | 3 (50.00) | 1 (16.67) | 1 (16.67) | 1 (16.67) | 0 (0.00) | 6 |
| 9. Mobility in community | 3–4 | 19 (18.27) | 36 (34.62) | 20 (19.23) | 16 (15.38) | 13 (12.50) | 104 |
|  | 5–6 | 49 (34.75) | 37 (26.24) | 34 (24.11) | 17 (12.06) | 4 (2.84) | 141 |
|  | 7–10 | 72 (37.70) | 48 (25.13) | 45 (23.56) | 21 (10.99) | 5 (2.62) | 191 |
|  | 11–14 | 39 (41.05) | 21 (22.11) | 20 (21.05) | 13 (13.68) | 2 (2.11) | 95 |
|  | 15–18 | 6 (42.86) | 4 (28.57) | 3 (21.43) | 1 (7.14) | 0 (0.00) | 14 |
|  | Missing | 2 (33.33) | 2 (33.33) | 2 (33.33) | 0 (0.00) | 0 (0.00) | 6 |
| 10.Communication in community | 3–4 | 45 (43.27) | 24 (23.08) | 14 (13.46) | 15 (14.42) | 6 (5.77) | 104 |
|  | 5–6 | 73 (51.77) | 27 (19.15) | 21 (14.89) | 15 (10.64) | 5 (3.55) | 141 |
|  | 7–10 | 103 (53.93) | 31 (16.23) | 27 (14.14) | 25 (13.09) | 5 (2.62) | 191 |
|  | 11–14 | 52 (54.74) | 16 (16.84) | 14 (14.74) | 12 (12.63) | 1 (1.05) | 95 |
|  | 15–18 | 8 (57.14) | 4 (28.57) | 0 (0.00) | 2 (14.29) | 0 (0.00) | 14 |
|  | Missing | 2 (33.33) | 3 (50.00) | 1 (16.67) | 0 (0.00) | 0 (0.00) | 6 |
| 11. Participation in classroom activities | 3–4 | 33 (31.73) | 31 (29.81) | 24 (23.08) | 14 (13.46) | 2 (1.92) | 104 |
|  | 5–6 | 62 (43.97) | 50 (35.46) | 21 (14.89) | 7 (4.96) | 1 (0.71) | 141 |
|  | 7–10 | 112 (58.64) | 37 (19.37) | 27 (14.14) | 15 (7.85) | 0 (0.00) | 191 |
|  | 11–14 | 55 (57.89) | 20 (21.05) | 10 (10.53) | 9 (9.47) | 1 (1.05) | 95 |
|  | 15–18 | 8 (57.14) | 4 (28.57) | 1 (7.14) | 1 (7.14) | 0 (0.00) | 14 |
|  | Missing | 3 (50.00) | 3 (50.00) | 0 (0.00) | 0 (0.00) | 0 (0.00) | 6 |
| 12. Social/leisure activities at school | 3–4 | 30 (28.85) | 40 (38.46) | 26 (25.00) | 8 (7.69) | 0 (0.00) | 104 |
|  | 5–6 | 45 (31.91) | 51 (36.17) | 34 (24.11) | 11 (7.80) | 0 (0.00) | 141 |
|  | 7–10 | 57 (29.84) | 64 (33.51) | 58 (30.37) | 12 (6.28) | 0 (0.00) | 191 |
|  | 11–14 | 40 (42.11) | 27 (28.42) | 20 (21.05) | 7 (7.37) | 1 (1.05) | 95 |
|  | 15–18 | 8 (57.14) | 3 (21.43) | 3 (21.43) | 0 (0.00) | 0 (0.00) | 14 |
|  | Missing | 2 (33.33) | 2 (33.33) | 2 (33.33) | 0 (0.00) | 0 (0.00) | 6 |
| 13. Mobility at school | 3–4 | 22 (21.15) | 42 (40.38) | 35 (33.65) | 5 (4.81) | 0 (0.00) | 104 |
|  | 5–6 | 45 (31.91) | 58 (41.13) | 34 (24.11) | 3 (2.13) | 1 (0.71) | 141 |
|  | 7–10 | 78 (40.84) | 71 (37.17) | 35 (18.32) | 7 (3.66) | 0 (0.00) | 191 |
|  | 11–14 | 37 (38.95) | 34 (35.79) | 20 (21.05) | 4 (4.21) | 0 (0.00) | 95 |
|  | 15–18 | 6 (42.86) | 6 (42.86) | 2 (14.29) | 0 (0.00) | 0 (0.00) | 14 |
|  | Missing | 3 (50.00) | 2 (33.33) | 1 (16.67) | 0 (0.00) | 0 (0.00) | 6 |
| 14. Use of learning materials | 3–4 | 42 (40.38) | 41 (39.42) | 16 (15.38) | 5 (4.81) | 0 (0.00) | 104 |
|  | 5–6 | 75 (53.19) | 42 (29.79) | 20 (14.18) | 3 (2.13) | 1 (0.71) | 141 |
|  | 7–10 | 121 (63.35) | 41 (21.47) | 21 (10.99) | 8 (4.19) | 0 (0.00) | 191 |
|  | 11–14 | 65 (68.42) | 14 (14.74) | 11 (11.58) | 5 (5.26) | 0 (0.00) | 95 |
|  | 15–18 | 8 (57.14) | 3 (21.43) | 2 (14.29) | 1 (7.14) | 0 (0.00) | 14 |
|  | Missing | 3 (50.00) | 3 (50.00) | 0 (0.00) | 0 (0.00) | 0 (0.00) | 6 |
| 15. Communication at school | 3–4 | 43 (41.35) | 34 (32.69) | 16 (15.38) | 6 (5.77) | 5 (4.81) | 104 |
|  | 5–6 | 71 (50.35) | 43 (30.50) | 19 (13.48) | 5 (3.55) | 3 (2.13) | 141 |
|  | 7–10 | 112 (58.64) | 48 (25.13) | 24 (12.57) | 7 (3.66) | 0 (0.00) | 191 |
|  | 11–14 | 51 (53.68) | 27 (28.42) | 10 (10.53) | 7 (7.37) | 0 (0.00) | 95 |
|  | 15–18 | 10 (71.43) | 2 (14.29) | 2 (14.29) | 0 (0.00) | 0 (0.00) | 14 |
|  | Missing | 4 (66.67) | 1 (16.67) | 1 (16.67) | 0 (0.00) | 0 (0.00) | 6 |
| 16. Household tasks | 3–4 | 32 (30.77) | 35 (33.65) | 27 (25.96) | 10 (9.62) | 0 (0.00) | 104 |
|  | 5–6 | 64 (45.39) | 51 (36.17) | 17 (12.06) | 9 (6.38) | 0 (0.00) | 141 |
|  | 7–10 | 111 (58.12) | 49 (25.65) | 25 (13.09) | 6 (3.14) | 0 (0.00) | 191 |
|  | 11–14 | 55 (57.89) | 24 (25.26) | 8 (8.42) | 6 (6.32) | 2 (2.11) | 95 |
|  | 15–18 | 8 (57.14) | 5 (35.71) | 1 (7.14) | 0 (0.00) | 0 (0.00) | 14 |
|  | Missing | 3 (50.00) | 3 (50.00) | 0 (0.00) | 0 (0.00) | 0 (0.00) | 6 |
| 17. Shopping and money use | 3–4 | 39 (37.50) | 28 (26.92) | 23 (22.12) | 10 (9.62) | 4 (3.85) | 104 |
|  | 5–6 | 62 (43.97) | 40 (28.37) | 24 (17.02) | 12 (8.51) | 3 (2.13) | 141 |
|  | 7–10 | 76 (39.79) | 65 (34.03) | 26 (13.61) | 23 (12.04) | 1 (0.52) | 191 |
|  | 11–14 | 35 (36.84) | 27 (28.42) | 23 (24.21) | 9 (9.47) | 1 (1.05) | 95 |
|  | 15–18 | 8 (57.14) | 3 (21.43) | 1 (7.14) | 2 (14.29) | 0 (0.00) | 14 |
|  | Missing | 3 (50.00) | 3 (50.00) | 0 (0.00) | 0 (0.00) | 0 (0.00) | 6 |
| 18. Planning daily routine | 3–4 | 17 (16.35) | 10 (9.62) | 2 (1.92) | 21 (20.19) | 54 (51.92) | 104 |
|  | 5–6 | 38 (26.95) | 12 (8.51) | 21 (14.89) | 24 (17.02) | 46 (32.62) | 141 |
|  | 7–10 | 59 (30.89) | 40 (20.94) | 32 (16.75) | 49 (25.65) | 11 (5.76) | 191 |
|  | 11–14 | 35 (36.84) | 21 (22.11) | 18 (18.95) | 19 (20.00) | 2 (2.11) | 95 |
|  | 15–18 | 7 (50.00) | 3 (21.43) | 1 (7.14) | 2 (14.29) | 1 (7.14) | 14 |
|  | Missing | 2 (33.33) | 1 (16.67) | 2 (33.33) | 0 (0.00) | 1 (16.67) | 6 |
| 19. Use of transportation | 3–4 | 14 (13.46) | 14 (13.46) | 9 (8.65) | 24 (23.08) | 43 (41.35) | 104 |
|  | 5–6 | 23 (16.31) | 31 (21.99) | 20 (14.18) | 32 (22.70) | 35 (24.82) | 141 |
|  | 7–10 | 29 (15.18) | 60 (31.41) | 42 (21.99) | 53 (27.75) | 7 (3.66) | 191 |
|  | 11–14 | 20 (21.05) | 25 (26.32) | 21 (22.11) | 27 (28.42) | 2 (2.11) | 95 |
|  | 15–18 | 6 (42.86) | 1 (7.14) | 3 (21.43) | 4 (28.57) | 0 (0.00) | 14 |
|  | Missing | 3 (50.00) | 0 (0.00) | 1 (16.67) | 2 (33.33) | 0 (0.00) | 6 |
| 20. Responsibility for daily activities | 3–4 | 19 (18.27) | 9 (8.65) | 3 (2.88) | 18 (17.31) | 55 (52.88) | 104 |
|  | 5–6 | 29 (20.57) | 14 (9.93) | 8 (5.67) | 28 (19.86) | 62 (43.97) | 141 |
|  | 7–10 | 62 (32.46) | 23 (12.04) | 23 (12.04) | 57 (29.84) | 26 (13.61) | 191 |
|  | 11–14 | 37 (38.95) | 13 (13.68) | 10 (10.53) | 25 (26.32) | 10 (10.53) | 95 |
|  | 15–18 | 9 (64.29) | 2 (14.29) | 1 (7.14) | 2 (14.29) | 0 (0.00) | 14 |
|  | Missing | 2 (33.33) | 1 (16.67) | 1 (16.67) | 1 (16.67) | 1 (16.67) | 6 |

n, absolute frequency; %, relative frequency.

Table S3. Descriptive statistics of Child and Adolescent Scale of Participation (CASP) scores by independent variables.

| **CASP scales**  **mean; median (min–max)** | **CASP total** | **Home** | **Community** | **School** | **Living** |
| --- | --- | --- | --- | --- | --- |
| Parental educational attainment – Medium and high | 76.08; 81 (25–100) | 78.80; 83 (25–100) | 75.66; 81 (25–100) | 78.81; 85 (25–100) | 69.92; 75 (25–100) |
| Parental educational attainment - Low | 75.73; 80 (25–100) | 79.37; 83 (25–100) | 76.05; 81 (25–100) | 79.74; 85 (25–100) | 65.98; 67 (25–100) |
| p-value^a^ | .58 | .95 | .72 | .86 | .08 |
| German native language | 76.03; 81 (25–100) | 79.03; 83 (25–100) | 75.85; 81 (25–100) | 79.62; 85 (25–100) | 68.19; 75 (25–100) |
| Non-native German language | 75.72; 80.50 (25–100) | 79.20; 83 (25–100) | 75.61; 81 (25–100) | 78.25; 85 (25–100) | 67.66; 72.50 (25–100) |
| p-value^a^ | .71 | .93 | .88 | .50 | .83 |
| Female | 75.69; 81 (25–100) | 78.80; 83 (25–100) | 74.61; 81 (25–100) | 79.41; 85 (25–100) | 68.16; 75 (25–100) |
| Male | 76.20; 81 (25–100) | 79.28; 83 (25–100) | 76.52; 81 (25–100) | 79.55; 85 (25–100) | 68.09; 70 (25–100) |
| p-value^a^ | .62 | .73 | .63 | .45 | .63 |
| Physical or cognitive impairment | 79.72; 81.50 (31–100) | 82.36; 83 (29–100) | 79.42; 81 (25–100) | 83.42; 85 (25–100) | 72.01; 75 (25–100) |
| Both impairments and others | 73.97; 80 (25–100) | 77.32; 83 (25–100) | 73.86; 81 (25–100) | 77.27; 85 (25–100) | 65.99; 70 (25–100) |
| p-value^a^ | .01 | .06 | .04 | <.01 | .01 |
| 3–6 years | 74.91; 80 (25–100) | 77.68; 83 (25–100) | 74.12; 81 (25–100) | 76.93; 80 (25–100) | 68.96; 75 (25–100) |
| 7–10 years | 76.63; 82 (25–100) | 80.43; 83 (25–100) | 77.00; 81 (25–100) | 81.17; 85 (25–100) | 66.49; 70 (25–100) |
| 11–18 years | 77.00; 81 (26–100) | 79.60; 83 (25–100) | 77.40; 88 (25–100) | 81.76; 90 (25–100) | 68.62; 70 (25–100) |
| p-value^b^ | .50 | .25 | .12 | <.01 | .38 |

Notes: Values are presented as: mean; median (minimum–maximum); CASP, Child and Adolescent Scale of Participation; Home, Community, School, and Living refer to CASP subscales. Missing values and responses answered as “not applicable” were not computed in CASP scores calculation. Higher scores thereby indicate more favourable participation. ^a^Mann–Whitney U test. ^b^Kruskal–Wallis test.

Table S4. Spearman's correlation coefficients - correlation matrix for independent variables.

|  | 1 | 2 | 3 | 4 | 5 | 6 |
| --- | --- | --- | --- | --- | --- | --- |
| 1. Parental educational attainment | 1.00 |  |  |  |  |  |
| 2. Parental German native language | -0.03 | 1.00 |  |  |  |  |
| 3.Child age | 0.08 | -0.17* | 1.00 |  |  |  |
| 4.Child sex | -0.01 | 0.06 | -0.00 | 1.00 |  |  |
| 5.Type of impairment | 0.10 | -0.04 | 0.00 | 0.12* | 1.00 |  |
| 6. HRQoL | -0.03 | 0.06 | -0.31* | -0.08 | -0.09 | 1.00 |

***,** p-value < .01; **, p-value = .05; children’s Health-related quality of life (HRQoL)

Table S5. Characteristics of children and families included in the regression models (complete-case sample, n = 350).

| Sample characteristics | Categories | n (%) / [mean (SD)] | Missing values |
| --- | --- | --- | --- |
| *Family characteristics* | | | |
| Parental educational attainment | High and Medium | 188 (53.71) | 0 |
|  | Low | 162 (46.28) |  |
| German native language | Yes | 305 (87.14) | 0 |
|  | No | 45 (12.85) |  |
| *Children characteristics* | | | |
| HRQoL |  | [64.16 (23.36)] | 0 |
| Age categories (as introduced into regression analysis) | 3–6 (Kindergarden) | 158 (45.14) | 0 |
|  | 7–10 (Elementary school) | 129 (36.85) |  |
|  | 11–18 (Secondary school) | 63 (18.00) |  |
| Sex | Female | 106 (30.28) | 0 |
|  | Male | 244 (69.71) |  |
| Primary clinical reason for referral to the SPCs | Physical or cognitive | 127 (36.28) | 0 |
|  | Both impairments and others | 223 (63.71) |  |
| *Social participation* | | | |
| CASP total score |  | [77.08 (19.21)] | 0 |
| Home subscale |  | [80.23 (19.61)] | 0 |
| Community subscale |  | [77.01 (22.24)] | 0 |
| School subscale |  | [80.44 (17.60)] | 0 |
| Living activities subscale |  | [69.31 (24.05)] | 0 |

CASP, Child and adolescent scale of social participation of children (0 from 100 scale, the higher is the score, the better is the social participation; HRQoL, health-related quality of life; continuous variables are expressed as means with standard deviations (SD), categorical variables are expressed as absolute (n) and relative frequencies (%);SPCs, Social Paediatric Centres. ^a^the highest educational attainment was assessed by the International Standard Classification of Education (ISCED), one parent filled out the item for both parents, and we took into account the highest level from both parents; ^b^ Other’ reasons refer to children without an established diagnosis at the time of consultation and/or whose condition was still under investigation.

Table S6. Univariable associations between characteristics of CSHCN and their families and social participation, Germany, 11/2018 and 01/2020.

| Variables | β | CI [lower limit; upper limit] | *p* | Mean VIF | R^2^ |
| --- | --- | --- | --- | --- | --- |
| *Total CASP scale* | | | | | |
| Parental educational attainment – Medium and high |  | Ref |  |  |  |
| Parental educational attainment - Low | -0.35 | [-4.21; 3.50] | .85 | 1.00 | 0.00 |
| German native language |  | Ref |  |  |  |
| Non-native German language | -0.31 | [-5.24; 4.61] | .90 | 1.00 | 0.00 |
| Female |  | Ref |  |  |  |
| Male | 0.51 | [-3.37; 4.39] | .79 | 1.00 | 0.00 |
| Physical or cognitive impairment |  | Ref |  |  |  |
| Both impairments and others | -5.75 | [-9.02; -2.48] | <.01 | 1.00 | 0.01 |
| HRQoL (parent) | 0.32 | [0.26; 0.39] | <.01 | 1.00 | 0.16 |
| Age - 3 to 4 years old |  | Ref |  |  |  |
| Age - 5 to 10 years old | 1.71 | [-2.03; 5.46] | .36 | 1.16 | 0.00 |
| Age - 11 to 18 years old | 2.09 | [-2.60; 6.78] | .38 | 1.16 | 0.00 |
| CASP subscales | | | | | |
| *Home* | | | | | |
| Parental educational attainment – Medium/high |  | Ref |  |  |  |
| Low | 0.56 | [-3.36; 4.49] | .77 | 1.00 | 0.00 |
| German native language |  | Ref |  |  |  |
| Non-native German | 0.16 | [-4.88; 5.22] | .94 | 1.00 | 0.00 |
| Female |  | Ref |  |  |  |
| Male | 0.48 | [-3.37; 4.34] | .80 | 1.00 | 0.00 |
| Physical/cognitive impairment |  | Ref |  |  |  |
| Both impairments and others | -5.03 | [-8.34; -1.72] | <.01 | 1.00 | 0.01 |
| HRQoL (parent) | 0.32 | [0.25; 0.39] | <.01 | 1.00 | 0.16 |
| Age 0–4 |  | Ref |  |  |  |
| Age 5–10 | 2.75 | [-1.01; 6.52] | .15 | 1.16 | 0.00 |
| Age 11–18 | 1.92 | [-2.81; 6.66] | .42 | 1.16 | 0.00 |
| *Community* | | | | | |
| Parental educational attainment – Medium/high |  | Ref |  |  |  |
| Low | 0.38 | [-4.02; 4.80] | .86 | 1.00 | 0.00 |
| German native language |  | Ref |  |  |  |
| Non-native German | -0.24 | [-6.08; 5.60] | .93 | 1.00 | 0.00 |
| Female |  | Ref |  |  |  |
| Male | 1.91 | [-2.39; 6.21] | .38 | 1.00 | 0.00 |
| Physical/cognitive impairment |  | Ref |  |  |  |
| Both impairments and others | -5.56 | [-9.35; -1.77] | <.01 | 1.00 | 0.01 |
| HRQoL (parent) | 0.34 | [0.26; 0.42] | <.01 | 1.00 | 0.14 |
| Age 0–4 |  | Ref |  |  |  |
| Age 5–10 | 2.87 | [-1.43; 7.19] | .19 | 1.16 | 0.00 |
| Age 11–18 | 3.28 | [-1.96; 8.52] | .21 | 1.16 | 0.00 |
| *School* | | | | | |
| Parental educational attainment – Medium/high |  | Ref |  |  |  |
| Low | 0.93 | [-2.64; 4.51] | .60 | 1.00 | 0.00 |
| German native language |  | Ref |  |  |  |
| Non-native German | -1.37 | [-6.15; 3.40] | .57 | 1.00 | 0.00 |
| Female |  | Ref |  |  |  |
| Male | 0.13 | [-3.48; 3.75] | .94 | 1.00 | 0.00 |
| Physical/cognitive impairment |  | Ref |  |  |  |
| Both impairments and others | -6.14 | [-9.15; -3.14] | <.01 | 1.00 | 0.02 |
| HRQoL (parent) | 0.25 | [0.19; 0.32] | <.01 | 1.00 | 0.12 |
| Age 0–4 |  | Ref |  |  |  |
| Age 5–10 | 4.23 | [0.75; 7.72] | .01 | 1.16 | 0.01 |
| Age 11–18 | 4.82 | [0.46; 9.18] | .03 | 1.16 | 0.01 |
| *Living Activities* | | | | | |
| Parental educational attainment – Medium/high |  | Ref |  |  |  |
| Low | -3.93 | [-8.69; 0.82] | .10 | 1.00 | 0.00 |
| German native language |  | Ref |  |  |  |
| Non-native German | -0.53 | [-6.70; 5.63] | .86 | 1.00 | 0.00 |
| Female |  | Ref |  |  |  |
| Male | -0.06 | [-4.90; 4.77] | .97 | 1.00 | 0.00 |
| Physical/cognitive impairment |  | Ref |  |  |  |
| Both impairments and others | -6.01 | [-10.32; -1.71] | .01 | 1.00 | 0.01 |
| HRQoL (parent) | 0.38 | [0.30; 0.45] | <.01 | 1.00 | 0.14 |
| Age 0–4 |  | Ref |  |  |  |
| Age 5–10 | -2.47 | [-7.11; 2.16] | .29 | 1.16 | 0.00 |
| Age 11–18 | -0.34 | [-6.07; 5.38] | .90 | 1.16 | 0.00 |

HRQoL, children’s Health-related quality of life; β, Standardized Regression Coefficient; VIF, Variance Inflation Factor; R-squared, coefficient of determination; Ref, reference category.
